# Expression of La Ribonucleoprotein Domain Family Member 4B (LARP4B) in Liver Cancer and Their Clinical and Prognostic Significance

**DOI:** 10.1155/2019/1569049

**Published:** 2019-10-22

**Authors:** Yanqing Li, Yan Jiao, Yang Li, Yanan Liu

**Affiliations:** ^1^Department of Pathophysiology, College of Basic Medical Sciences, Jilin University, Changchun, Jilin 130021, China; ^2^Department of Hepatobiliary and Pancreatic Surgery, The First Hospital of Jilin University, Changchun, Jilin 130021, China

## Abstract

**Background and Objective:**

Liver cancer is a common malignant tumor with few poor diagnostic and prognostic markers, which greatly shortens the potential life span of patients. The RNA-binding protein la ribonucleoprotein 4B (LARP4B) has a la motif (lam) that is important in the process of cancer. We aimed to explore the role of LARP4B in the diagnosis and prognosis of liver cancer.

**Methods:**

The Cancer Genome Atlas (TCGA) database was searched to detect *LARP4B* gene expression in liver cancer. The clinical relevance and diagnostic ability of LARP4B were evaluated by a chi-squared test and a receiver operating characteristic (ROC) curve, respectively. Survival and risk factors of patients with liver cancer were assessed by survival analysis and univariate/multivariate Cox regression model. Additionally, we carried out gene set enrichment analysis (GSEA) to identify LARP4B-related signaling pathways in liver cancer.

**Results:**

*LARP4B* mRNA was highly expressed in liver cancer tissues and was correlated with survival status. The chi-squared test showed that LARP4B had clinical relevance, while ROC curves showed that LARP4B had good diagnostic ability. Survival analysis showed that liver cancer patients with high *LARP4B* expression had shorter overall/relapse-free survival. The univariate/multivariate Cox regression model indicated that high *LARP4B* expression may be an independent risk factor for the prognosis of liver cancer patients. Finally, we found that genes involved in the G2M checkpoint, E2F targets, and mitotic spindle were differentially enriched in the high *LARP4B*-expression phenotype.

**Conclusions:**

LARP4B is a potential independent biomarker for diagnosis and prognosis in liver cancer patients.

## 1. Introduction

Liver cancer is one of the most common malignant tumors in the world [1]. It has a high degree of malignancy, strong invasion and metastasis, and poor prognosis and poses a serious threat to health [2]. Although the number of tumor markers used in the diagnosis and prognosis of related cancers has increased, none have the high recognition, sensitivity, and specificity required to evaluate the condition, efficacy, and prognosis of liver cancer [3, 4]. There is an urgent need to identify biomarkers with diagnostic and prognostic accuracy.

La ribonucleoprotein 4B (LARP4B) is a member of the La-related protein (LARP) family [5]. LARP4B protein is an RNA-binding protein containing lanthanum and adjacent RNA recognition motifs (RRMs) [6], which allow it to participate in posttranscriptional control of RNA and play an important role in translation [7, 8]. LARP4B is involved in the progression of many cancers [9]. In gliomas, for example, LARP4B inhibits tumor progression [10]. However, its role in liver cancer has not been explored.

To assess the potential clinical role of LARP4B in liver cancer, we probed TCGA database for the mRNA expression of *LARP4B* in liver cancer patients. Chi-squared testing was used to assess clinical relevance, ROC curves were used to estimate diagnostic capability, and overall/relapse-free survival analyses were conducted to examine the impact of LARP4B on patients with liver cancer. Univariate/multivariate Cox regression models were used to identify risk factors associated with liver cancer. We also carried out GSEA about the LARP4B-related signaling pathways.

## 2. Materials and Methods

### 2.1. Data Source

We obtained currently available clinical and RNA sequence data about normal and liver cancer tissues from TCGA (https://cancergenome.nih.gov/). No ethical permission was required because all of the data used in this paper were made available for research.

### 2.2. Data Mining and Statistical Analyses

We used the R software environment (version 3.6.1) for data mining [11]. Boxplots of clinical features were drawn with the ggplot2 package [12]. The ROC curve was drawn by pROC [13], which is based on a series of different binary classifications (demarcation value or determination threshold), plotting the true positive rate (sensitivity) as the ordinate and the false positive rate (1-specificity) as the abscissa. Among them, the area under the ROC curve (AUC) is used to measure the diagnostic performance. The chi-squared test was used to identify possible clinical correlations between clinical features and *LARP4B* expression. We also used survival packages to plot survival curves, and a logarithmic rank test to check survival bias [14]. Univariate and multivariate Cox models were used to distinguish risk factors associated with liver cancer [15].

### 2.3. GSEA

GSEA is used to classify gene probes based on related biological pathways published in authoritative journals and coexpression data obtained from experiments. To determine correlation, a series of operations are carried out to determine whether the probes can reveal a distribution pattern of genes related to the phenotype of interest [16]. In this research, we used the gene set of “h.all.v6.2.symbols.gmt” from the Molecular Signatures Database to perform GSEA in GSEA 3.0 software. Through the analysis of 1,000 permutations, we obtained the standardized enrichment fraction and calculated a normalized enrichment score.

## 3. Results

### 3.1. Data Overview

The *LARP4B* expression level and clinical features of the TCGA liver cancer cohort include gender, age, histologic type and grade, sample type, T/N/M classification, radiation therapy, residual tumor, vital status, stage, and relapse (Table 1).

### 3.2. *LARP4B* Expression in Normal and Liver Cancer Tissues

**Figure 1:**
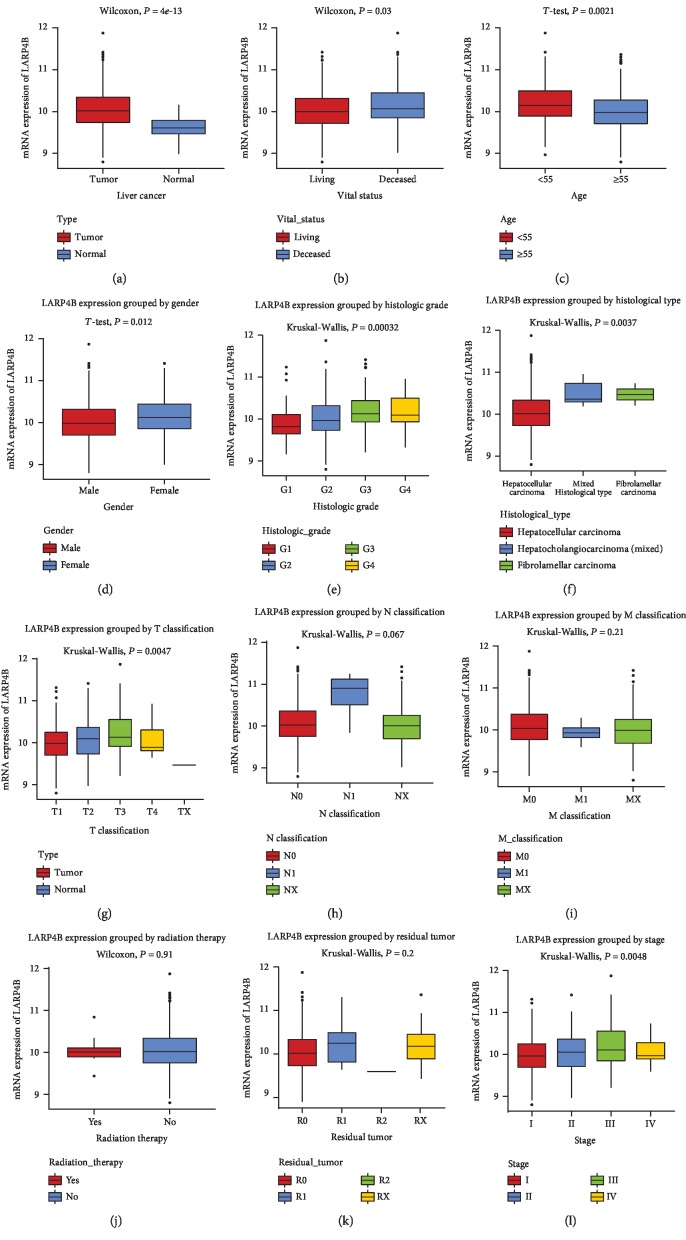
Boxplots showing *LARP4B* expression according to clinical stage and tissue type.

Boxplots showed higher *LARP4B* mRNA expression in liver cancer tissues compared with normal liver tissues (*P* = 4*e*‐13; Figure 1). Furthermore, there were significant differences in *LARP4B* expression with regard to vital status (*P* = 0.03), stage (*P* = 0.0046), gender (*P* = 0.012), age (*P* = 0.0021), histologic grade (*P* = 0.00032), type (*P* = 0.0037), and T classification (*P* = 0.047).

### 3.3. Diagnostic Capability of *LARP4B* in Liver Cancer

**Figure 2:**
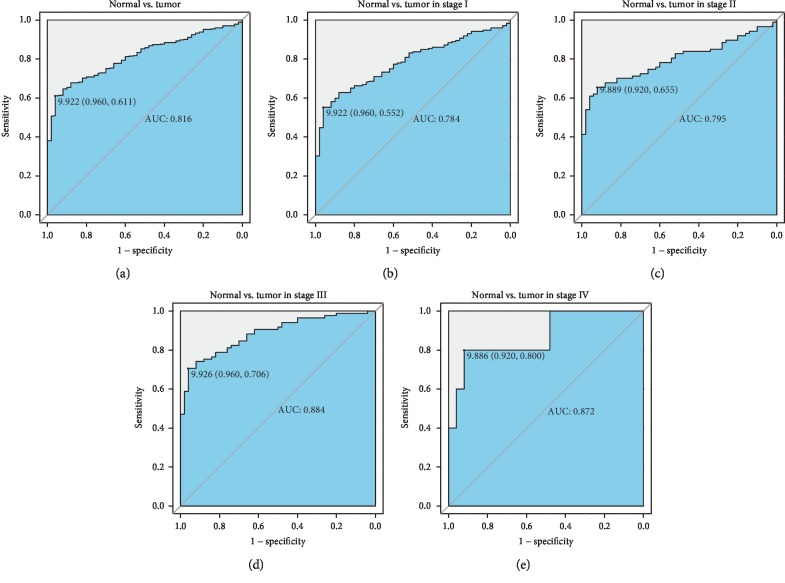
ROC curves of LARP4B expression in the TCGA-LIHC cohort. Normal liver vs. liver tumor samples (a). Normal liver vs. stage I (b), stage II (c), stage III (d), and stage IV (e) liver tumor samples. AUC: area under the curve; LIHC: liver hepatocellular carcinoma; ROC: receiver operating characteristic curve.

ROC curves revealed that AUC was 0.816, indicating that *LARP4B* might have considerable diagnostic ability (Figure 2(a)). This result was confirmed in subsequent subgroup analysis of the different stages (AUC: 0.784, 0.795, 0.884, and 0.872 for stage I, stage II, stage III, and stage IV, respectively; Figures 2(b)–2(e)).

### 3.4. High *LARP4B* Expression Was Relevant to Clinical Features of Liver Cancer

As shown in Table 2, the expression of *LARP4B* was clearly related to age (*P* = 0.0274), gender (*P* = 0.0256), vital status (*P* = 0.0301), and histologic grade (*P* = 0.0003) of liver cancer patients.

### 3.5. Increased *LARP4B* Expression Was Related to Poor Overall Survival in Liver Cancer

**Figure 3:**
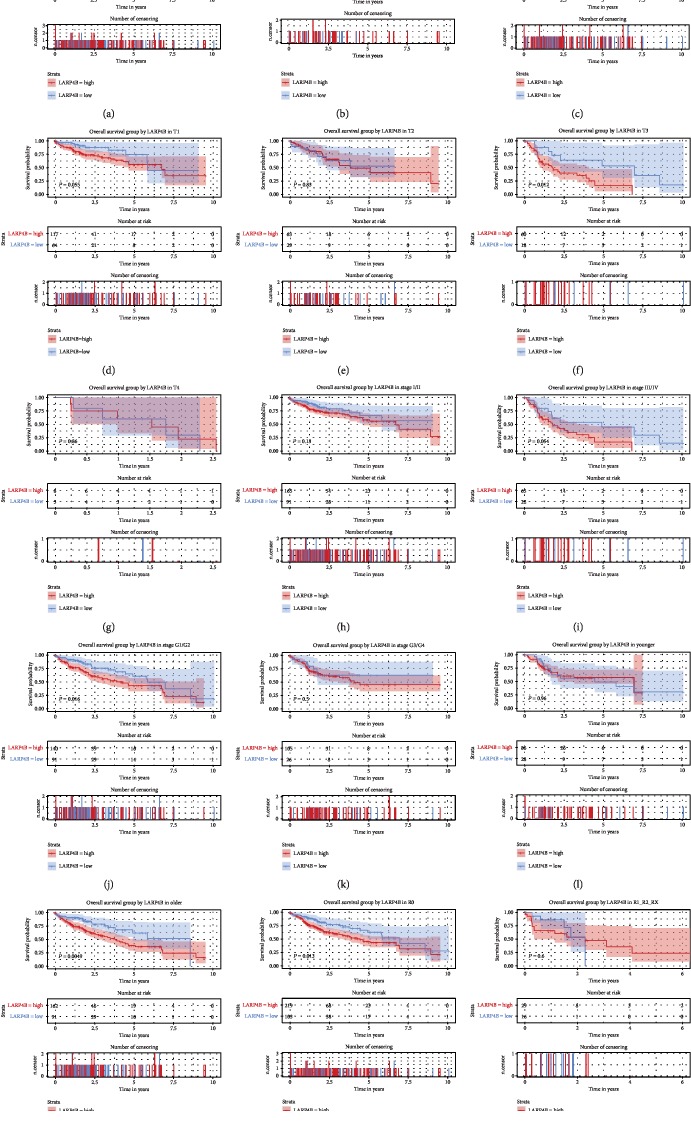
Effect of *LARP4B* expression on overall survival in subgroups of patients with liver cancer. Kaplan-Meier curves of overall survival analysis (a) and subgroup analysis of gender (female and male) (b, c), T classification (T1/T2/T3/T4) (d–g), clinical stage (I/II and III/IV) (h, i), histologic grade (G1/G2 and G3/G4) (j, k), age (younger and older) (l, m), and lymph node dissection (R0 and R1/R2/RX) (n, o).

As shown in Figure 3, the high expression of *LARP4B* in patients was correlated with poor overall survival (*P* = 0.0095). Subgroup analysis showed that *LARP4B* expression had significant prognostic value in liver cancer patients who were older (*P* = 0.0049), T3 (*P* = 0.012), G1/G2 (*P* = 0.016), male (*P* = 0.01), and R0 (*P* = 0.013).

A univariate Cox model revealed that residual tumor, stage, T classification, and *LARP4B* expression represented potential survival-related variables. A multivariate Cox model suggested that a high *LARP4B* expression was a potential independent risk factor for patient's overall survival with liver cancer (95% confidence interval (CI) 1.1–2.46, *P* = 0.016, hazard ratio (HR) = 1.64; Table 3).

### 3.6. Increased *LARP4B* Expression Was Related to Poor Relapse-Free Survival in Liver Cancer

**Figure 4:**
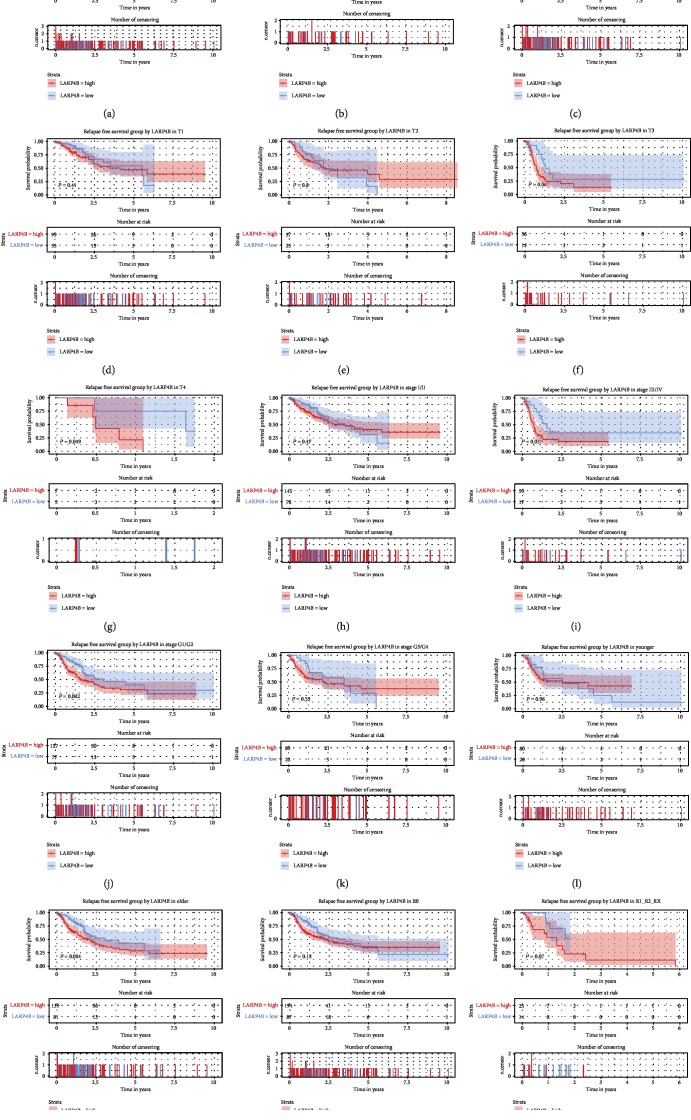
Effect of *LARP4B* expression on relapse-free survival in subgroups of patients with liver cancer. Kaplan-Meier curves of overall survival analysis (a) and subgroup analysis of gender (female and male) (b, c), T classification (T1/T2/T3/T4) (d–g), clinical stage (I/II and III/IV) (h, i), histologic grade (G1/G2 and G3/G4) (j, k), age (younger and older) (l, m), and lymph node dissection (R0 and R1/R2/RX) (n, o).

As shown in Figure 4, there was a high expression of *LARP4B* in patients with relapse-free survival (*P* = 0.044). Subgroup analysis showed that *LARP4B* expression had a prognostic value in liver cancer patients who were T4 (*P* = 0.049), male (*P* = 0.014), stage III/IV (*P* = 0.037), G1/G2 (*P* = 0.022), and older (*P* = 0.024).

A univariate Cox model showed that residual tumor, stage, T classification, and *LARP4B* expression represented potential relapse-free survival-related variables. A multivariate Cox model suggested that a high *LARP4B* expression was a potential independent risk factor for relapse-free survival in liver cancer patients (95% CI 1–2.13, *P* = 0.048, HR = 1.46; Table 4).

### 3.7. LARP4B-Related Signaling Pathway

**Figure 5:**
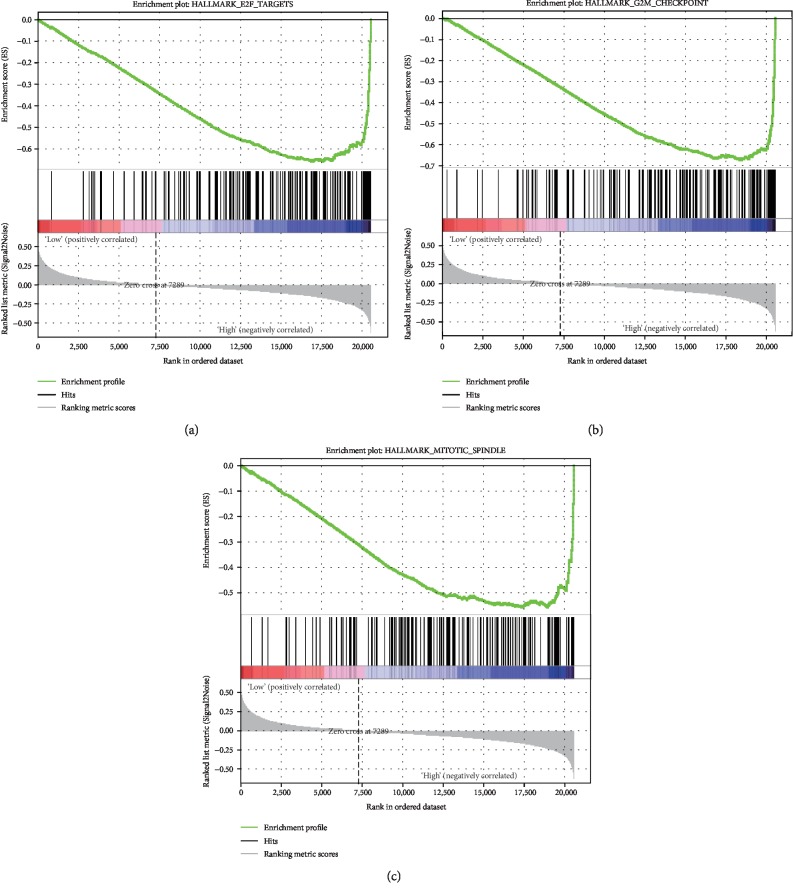
Enrichment plots from GSEA. E2F targets, the G2M checkpoint, and the mitotic spindle pathway were differentially enriched in LARP4B-related liver cancer patients.

To identify the activated signal pathways in liver cancer, GSEA was conducted between the low *LARP4B* expression and high *LARP4B* expression datasets. GSEA revealed significant differences in the enrichment of the MSigDB Collection (h.all.v6.2.symbols.gmt; NOM *P* value < 0.05, FDR < 0.25; Table 5). Genes related to E2F, G2M, and the mitotic spindle (Figure 5; Table 5) were enriched in the high LARP4B expression phenotype, which may represent an intrinsic mechanism of poor prognosis.

## 4. Discussion

Viral infection, diet, environmental problems, and other factors have contributed to the high mortality rate of liver cancer worldwide [17]. Continuous advances in surgical technology, chemotherapeutic drugs, and molecular biology have furthered our understanding of cancer biology, and there has been great progress in the treatment of liver cancer in recent years. In this study, we have applied our extensive experience in the exploration of novel biomarkers [18–23] to identify a biomarker, LARP4B, for the diagnosis and prognosis of patients with liver cancer.

The involvement of LARP4B has been documented in many cancer processes, including medulloblastoma [24], malignant peripheral nerve sheath tumors [25], colorectal cancer [26], pancreatic cancer [27], and glioma [10]. In contrast to our results, Koso et al. [10] found low expression of LARP4B in gliomas, which suggests that LARP4B may play different roles in different cancers. In addition, the boxplots showed that *LARP4B* expression was statistically significantly associated with vital status, age, gender, histologic grade and type, stage, and T classification. Therefore, it is necessary to further explore the role of LARP4B in liver cancer.

Low LARP4B expression was closely related to poor prognosis in glioma cancer patients in the study by Koso et al. [10]. No such relationship between LARP4B and prognosis has been found in liver cancer. In this study, we found that overexpressed *LARP4B* was associated with a poor prognosis in liver cancer patients, which may be attributable to the different functions of LARP4B in different tissues. LARP4B overexpression also led to shorter overall/relapse-free survival. Subgroup analysis of overall survival showed especially poor prognoses in patients who were male, T3, G1/G2, older, and R0, while relapse-free survival was correlated with patients who were male, T4, G1/G2, older, and stage III/IV. This distinction could be applied to the precise, individualized treatment of liver cancer patients. Calculation of AUC from the ROC curves showed the validity of clinical diagnostic testing of LARP4B. Our results suggest that LARP4B has strong potential as a marker in the clinical detection of liver cancer patients.

The progression of cancer requires a complete cell cycle [28]. Genes related to E2F, G2M, and the mitotic spindle are important signaling pathways in the cell cycle [29–31]. Interestingly, we found that E2F, G2M, and mitotic spindle signaling pathways were all involved in the progression of liver cancer. LARP4B is a posttranscriptional regulator and may regulate the roles of downstream genes through these three sets of target molecules. In addition, Zhang et al. [32] found that LARP4B may regulate the cell cycle of leukemia stem cells by inhibiting the expression of cell cycle inhibitors p16, P19, and p21 and myeloid-specific transcription factor CCAAT enhancer binding protein alpha. However, Mattijssen and Maraia [33] found that LARP4B participated in the regulation of TNF-alpha-TTP as its functional activity in MLL-AF9 leukemia stem cells. It is possible that LARP4B participates in the progression of different cancers through multiple signaling pathways.

As far as we know, this is the first study to examine the diagnostic and prognostic values of *LARP4B* expression in liver cancer. Together with other studies on the functions of LARP4B, we have contributed to a better understanding of the role of LARP4B and expanded the possibilities for more precise diagnosis and prognosis in cancer. We plan to continue exploring the functions of LARP4B to clarify its underlying mechanism in tumorigenesis at a deeper level.

## 5. Conclusion

In this investigation of LARP4B in the prognosis and diagnosis of liver cancer, we identified high LARP4B expression as a potential independent biomarker for negative prognosis. We have plans for complex experiments to explore the mechanism further.

## Figures and Tables

**Table 1:** Demographic and clinical characteristics of the TCGA-LIHC cohort.

Characteristics	Number of cases (%)
Age	
<55	117 (31.45)
≥55	255 (68.55)
NA	1 (0)
Gender	
Female	121 (32.44)
Male	252 (67.56)
Histological type	
Fibrolamellar carcinoma	3 (0.8)
Hepatocellular carcinoma	363 (97.32)
Hepatocholangiocarcinoma (mixed)	7 (1.88)
Histologic grade	
NA	5 (1.34)
G1	55 (14.75)
G2	178 (47.72)
G3	123 (32.98)
G4	12 (3.22)
Stage	
NA	24 (6.43)
I	172 (46.11)
II	87 (23.32)
III	85 (22.79)
IV	5 (1.34)
T classification	
NA	2 (0.54)
T1	182 (48.79)
T2	95 (25.47)
T3	80 (21.45)
T4	13 (3.49)
TX	1 (0.27)
N classification	
NA	1 (0.27)
N0	253 (67.83)
N1	4 (1.07)
NX	115 (30.83)
M classification	
M0	267 (71.58)
M1	4 (1.07)
MX	102 (27.35)
Radiation therapy	
NA	25 (6.7)
No	340 (91.15)
Yes	8 (2.14)
Residual tumor	
NA	7 (1.88)
R0	326 (87.4)
R1	17 (4.56)
R2	1 (0.27)
RX	22 (5.9)
Vital status	
Deceased	130 (34.85)
Living	243 (65.15)
Sample type	
Primary tumor	371 (99.46)
Recurrent tumor	2 (0.54)
LARP4B	
High	253 (67.83)
Low	120 (32.17)

Abbreviation: NA: not available.

**Table 2:** Correlation between the expression of LARP4B and the clinic pathologic characteristics in liver cancer.

Clinical characteristics	Variable	No. of patients	LARP4B expression				*X* ^2^	*P*
			High	%	Low	%		
Age	<55	117	89	35.32	28	23.33	4.8736	0.0273
	≥55	255	163	64.68	92	76.67		
Gender	Female	121	92	36.36	29	24.17	4.9824	0.0256
	Male	252	161	63.64	91	75.83		
Histological type	Fibrolamellar carcinoma	3	3	1.19	0	0	4.8737	0.0874
	Hepatocellular carcinoma	363	243	96.05	120	100		
	Hepatocholangiocarcinoma (mixed)	7	7	2.77	0	0		
Histologic grade	G1	55	27	10.8	28	23.73	18.9592	0.0003
	G2	178	115	46	63	53.39		
	G3	123	98	39.2	25	21.19		
	G4	12	10	4	2	1.69		
Stage	I	172	108	45.96	64	56.14	4.4368	0.218
	II	87	59	25.11	28	24.56		
	III	85	64	27.23	21	18.42		
	IV	5	4	1.7	1	0.88		
T classification	T1	182	117	46.25	65	55.08	6.9572	0.1382
	T2	95	66	26.09	29	24.58		
	T3	80	62	24.51	18	15.25		
	T4	13	8	3.16	5	4.24		
	TX	1	0	0	1	0.85		
N classification	N0	253	174	69.05	79	65.83	2.6345	0.2679
	N1	4	4	1.59	0	0		
	NX	115	74	29.37	41	34.17		
M classification	M0	267	188	74.31	79	65.83	3.2303	0.1989
	M1	4	3	1.19	1	0.83		
	MX	102	62	24.51	40	33.33		
Radiation therapy	No	340	233	97.08	107	99.07	0.5773	0.4474
	Yes	8	7	2.92	1	0.93		
Residual tumor	R0	326	223	88.49	103	90.35	2.9922	0.3928
	R1	17	12	4.76	5	4.39		
	R2	1	0	0	1	0.88		
	RX	22	17	6.75	5	4.39		
Sample type	Primary tumor	371	252	99.6	119	99.17	0	1
	Recurrent tumor	2	1	0.4	1	0.83		
Vital status	Deceased	130	98	38.74	32	26.67	4.7032	0.0301
	Living	243	155	61.26	88	73.33		

**Table 3:** Summary of univariate and multivariate Cox regression analyses of overall survival duration.

Parameters	Univariate analysis			Multivariate analysis		
	Hazard ratio	95% CI (lower~upper)	*P* value	Hazard ratio	95% CI (lower-upper)	*P* value
Age	1	0.69-1.45	0.997			
Gender	0.8	0.56-1.14	0.22			
Histological type	0.99	0.27-3.66	0.986			
Histologic grade	1.04	0.84-1.3	0.698			
Stage	1.38	1.15-1.66	0.001	0.87	0.7-1.08	0.203
T classification	1.66	1.39-1.99	0	1.85	1.46-2.34	0
N classification	0.73	0.51-1.05	0.086			
M classification	0.72	0.49-1.04	0.077			
Radiation therapy	0.51	0.26-1.03	0.06			
Residual tumor	1.42	1.13-1.8	0.003	1.39	1.08-1.78	0.01
LARP4B	1.69	1.13-2.52	0.01	1.64	1.1-2.46	0.016

**Table 4:** Summary of univariate and multivariate Cox regression analyses of relapse-free survival duration.

Parameters	Univariate analysis			Multivariate analysis		
	Hazard ratio	95% CI (lower~upper)	*P* value	Hazard ratio	95% CI (lower~upper)	*P* value
Age	0.9	0.63-1.28	0.55			
Gender	0.99	0.7-1.41	0.966			
Histological type	2.02	0.66-6.24	0.22			
Histologic grade	0.98	0.8-1.21	0.883			
Stage	1.66	1.38-1.99	0	1.12	0.87-1.44	0.392
T classification	1.78	1.49-2.12	0	1.67	1.28-2.17	0
N classification	0.97	0.67-1.4	0.874			
M classification	1.17	0.79-1.74	0.432			
Radiation therapy	0.74	0.26-2.16	0.584			
Residual tumor	1.28	1.01-1.61	0.042	1.31	1.03-1.67	0.026
LARP4B	1.46	1.01-2.11	0.045	1.46	1-2.13	0.048

**Table 5:** Gene sets enriched in phenotype high.

MSigDB collection	Gene set name	NES	NOM *p* val	FDR *q* val
h.all.v6.2.symbols.gmt	HALLMARK_MITOTIC_SPINDLE	-1.846927	0.005917	0.205141
h.all.v6.2.symbols.gmt	HALLMARK_G2M_CHECKPOINT	-1.710802	0.016293	0.255083
h.all.v6.2.symbols.gmt	HALLMARK_E2F_TARGETS	-1.583288	0.045643	0.40425

Notes: gene sets with NOM *P* value < 0.05 and FDR *q* value < 0.25 are considered as significant. Abbreviations: FDR: false discovery rate; NES: normalized enrichment score; NOM: nominal.

## Data Availability

We obtained patient information from an open TCGA database. No private clinical studies or patient data were included in this study.
